# Insights into Shared Decision-Making in Interprofessional Teams for a Boy with Down Syndrome with Communication and Language Issues: Simulation-Based Training for Medical and Allied Health Students

**DOI:** 10.3390/healthcare12060681

**Published:** 2024-03-18

**Authors:** Stijn R. J. M. Deckers, Yvonne van Zaalen

**Affiliations:** 1 Stichting Milo, Center for Augmentative and Alternative Communication, 5482 JH Schijndel, The Netherlands; 2 Department of Pedagogical Sciences, Radboud University, 6525 XZ Nijmegen, The Netherlands; 3 Research Group Relational Care, Knowledge Center Health Innovation, The Hague University of Allied Sciences, 2521 EN The Hague, The Netherlands; y.vanzaalen@hhs.nl

**Keywords:** shared decision-making, interprofessional collaboration, group dynamics, interprofessional communication, Down syndrome

## Abstract

Background: Shared decision-making is one key element of interprofessional collaboration. Communication is often considered to be the main reason for inefficient or ineffective collaboration. Little is known about group dynamics in the process of shared decision-making in a team with professionals, including the patient or their parent. This study aimed to evaluate just that. Methods: Simulation-based training was provided for groups of medical and allied health profession students from universities across the globe. In an overt ethnographic research design, passive observations were made to ensure careful observations and accurate reporting. The training offered the context to directly experience the behaviors and interactions of a group of people. Results: Overall, 39 different goals were defined in different orders of prioritizing and with different time frames or intervention ideas. Shared decision-making was lacking, and groups chose to convince the parents when a conflict arose. Group dynamics made parents verbally agree with professionals, although their non-verbal communication was not in congruence with that. Conclusions: The outcome and goalsetting of an interprofessional meeting are highly influenced by group dynamics. The vision, structure, process, and results of the meeting are affected by multiple inter- or intrapersonal factors.

## 1. Introduction

### 1.1. Interprofessional Collaboration

The World Health Organization considers the ability of professionals to work together effectively and efficiently as one of the greatest challenges to improving health care and relief efforts worldwide. This is particularly true of societies with a growing population of older people. The Netherlands is an example of this. Professionals from multiple disciplines are often involved in the care and support of a particular client. It is in the interest of the client and his health that the professionals involved work in a coordinated manner. This sounds obvious, but practice shows that working together is not always easy. That is why the World Health Organization is calling for more interprofessional training for all health professionals. The idea is that an interprofessionally trained health worker can work better in professional practice with colleagues from other disciplines. Additionally, better collaboration increases the chance of better health outcomes for the client [1]. The same plea can be made for the training of professionals working in the social domain or education.

Van Zaalen et al. mention that prerequisites for achieving good, efficient, and effective interprofessional cooperation are knowledge of each other’s treatment options, knowledge of each other’s information needs, and the use of each other’s language [2], as expressed, for example, in the internationally used International Classification of Functioning, Disability, and Health (ICF) and the children and youth version (ICF-CY). In the present article, we focus on the ICF-CY. The difference with the ICF is that the ICF-CY not only focusses on medical parameters but takes a more developmental perspective in that learning processes in the developing child can be indicated. The ICF-CY is a classification framework that can be used by all disciplines in health and social care to describe human functioning and participation. As we all know, working well together can sometimes be quite difficult. If professionals want to work together effectively and efficiently, it is necessary that they are prepared to do the following [3]:

- step out of the boundaries of their own discipline;
- discover new areas of knowledge and experience;
- learn to understand another (professional) language (and culture);
- accommodate multiple perspectives and points of view of different professionals and clients;
- make room for the expertise of a colleague;
- value the expertise and vision of a colleague as complementary and enriching.

We see the development of collaboration in care, welfare, and education, in which, as a result of increasingly complex care and support issues, more and more disciplines are involved and have to work together. Additionally, the role, involvement, and responsibility of the client is growing. As a result, collaboration also has other characteristics:

- Vision. There will be more emphasis on ‘the whole person’ and the context in which the person functions, e.g., a holistic view of health and participation. Multiple perspectives are important, and multiple factors and their influences on health and well-being play a role.
- Structure. The complexity of care demands is increasing, and cooperation between different disciplines is intensifying. Hierarchical positions and clearly defined tasks and roles become less important.
- Processes. Communication skills are becoming increasingly important since more frequent and different interactions with both professionals and clients are occurring. This requires different skills to achieve synergy and to reach a consensus in determining intervention options and goals. Clients are increasingly in control. Care, support, and guidance are increasingly tailored to the needs of specific people and their environment. The autonomy of the individual professional is reduced. Shared decision-making with the client and their social network is considered to be standard.
- Results. The complexity and diversity of possible outcomes increases. Next to this, outcomes of care and interventions are not merely measured as decreasing health issues but as increasing the level of functioning, participation, and inclusion.

### 1.2. Shared Decision-Making

In the present study, we will focus more on shared decision-making in the interprofessional context. Shared decision-making, a process whereby health professionals and patients work together to make healthcare choices, is fundamental to informed consent and patient-centered care [4,5]. Patient-centered care, client-centered care, and shared decision-making are three definitions for approaches that mean the same thing or are closely related. In Dutch, people often talk about ‘deciding together’. Since January 2020, shared decision-making has been laid down as conditional in Dutch law. According to the WGBO (Medical Treatment Agreement Act), it is the duty of the professional to make a shared decision. This also applies to offering the option to refrain from a (medical) intervention and to investigate the client’s preferences together. Despite this obligation, it is still seen that Dutch guidelines pay little attention to shared decision-making [6], which also seems to be the case for other countries. Shared decision-making can only yield results if the communication between the client, a potentially involved partner from the social network, and various professionals is effective and efficient. Ineffective communication often causes the client to participate less actively in the decision-making process [7], which is directly related to reduced health outcomes, increased risk of errors in both diagnosis and treatment [8], lower quality of care, and lower client satisfaction with the support provided [9].

Shared decision-making is a constant challenge, especially in an interprofessional context. Sharing knowledge and experiences during a decision-making meeting does not guarantee that the team members will continue to work together afterwards [10]. Each team member will make their own personal interpretation of the situation based on the information provided by the others and then make their own prioritization with regard to the steps in the care and support process. This means that professionals must be able to deal with perspectives from the client himself, from his social network, and from other professionals, and they must adjust their own perspectives accordingly. Only when a common perspective has been reached through communication, with agreement on the prioritization of advice, can we really speak of shared decision-making in the interprofessional context. Motivating and challenging a team to discuss conflicting ideas and perspectives (including those of the client and their social network) and to transform them into a single perspective will benefit the client’s care, education, and support.

In the field of shared decision-making, low health literacy, with a strong relationship to literacy skills, is considered to be one of the greatest potential risks to good, shared decision-making. Although shared decision-making is complex enough, this is even more so if the client has limited health skills. Of the Dutch adult population, 25 percent have limited health literacy [11]. Low literacy means that people have a poor command of the language(s) they speak. For example, they have difficulty with reading, writing, or arithmetic and with understanding complex language. As a result, their health literacy will be limited as well, i.e., they find it difficult to obtain, understand, assess, and use information about illness, health, well-being, education, and upbringing when making decisions for themselves or for others, for example, for their children. In the Netherlands, this concerns approximately 2.5 million people.

It is important for professionals to be aware of low literacy and limited health literacy and to recognize the manifestations of this so that they can better tailor their communication to the communicative possibilities that people do have. Simplifying information is thus necessary but not sufficient for the active participation of the client and his social networks in the decision-making process. Muscat et al. describe what it takes to successfully participate in shared decision-making [12]. Clients need skills in communicating effectively and in obtaining, understanding, and sharing information with both professionals and stakeholders from their social network. Clients also need cognitive and social skills to be able to share their own values, preferences, and previous experiences, as well as to be able to evaluate the information given by the professionals, apply it to their own situation, and test it against their own values and preferences. Therefore, it is important for shared decision-making to have insight into the client’s health literacy. Does the client really have all the information? Additionally, can he make a link to his own situation, values, and preferences?

All these challenges and skills needed in shared decision-making in interprofessional collaboration require different training and education methods for professionals based on real-life cases. For the present study, we focused on shared decision-making for a child with Down syndrome.

### 1.3. Communication and Language Development in Down Syndrome

Children with Down syndrome (DS) often present with intellectual disabilities, visual impairments, and hearing loss, are typically delayed in learning to speak, and, in the first years of life, make use of vocalizations and gestures [13]. Even after they begin to speak, these children may be difficult to understand [14]. This poor intelligibility and the significantly delayed onset of speech are two primary reasons for introducing augmentative and alternative communication (AAC) to children with DS [13]. AAC entails all strategies, tools, and communication forms that can be used to support communication and language development. For example, looking-and-pointing behaviors, communication books, manual signs, speech-generating devices, and tables with pictograms. Given the wide array of possibilities in AAC and the need to match the needs and strengths of the child to the characteristics of AAC strategies and tools [15], it is a clinical challenge to disentangle various mechanisms that contribute to the language and communication problems that children with DS encounter, which makes this topic a relevant case for interprofessional collaboration and shared decision-making.

The research on decision-making in AAC assessment has been limited, and the extant literature focuses mainly on decisions regarding the design of communication displays (e.g., [16]). The ultimate goal of AAC should be to enable the child with DS to efficiently and effectively engage in various interactions and participate in activities of their choice [17]. Therefore, it is of great importance to identify the skills and opportunities of children with DS since these can work as enablers or facilitators for the child and its important social partners [18]. This outline of a child’s skills and needs in relation to specific adaptations in its natural environment should guide clinical decision-making through AAC assessment and intervention planning [17]. Therefore, it is not sufficient to characterize a child using the label Down Syndrome alone, but it is necessary to describe the impact of the syndrome on various developmental domains for the individual child [19]. Meisels and Atkins-Burnett [20] state that to consider only one domain of development in isolation from others leaves the influence of the other domains unrecognized and “may obscure our understanding of the child’s abilities and challenges” (p. 232). Without clarity of different levels of functioning, appropriate interventions may be poorly conceived or improperly implemented. Successful AAC implementation requires an integrated perspective, which can be based on the conceptual framework and taxonomy of the ICF-CY [21].

Such a strengths-based approach that emphasizes an individual’s strengths and skills is critical in AAC assessment because these strengths can then be matched to one or more AAC techniques [17]. Still, little is known about how practitioners make decisions in AAC, but it could be speculated that most decisions are based on practitioner familiarity, clinical reasoning from experience, practices promoted in continuing education/professional development activities, discussions with colleagues, and some use of research evidence [22]. Furthermore, decision-making in AAC has been described as being too child-focused and not family-focused enough [23]; although, in planning AAC interventions, families should have the opportunity to provide input regarding what they perceive to be their children’s greatest needs and their own highest priorities [24]. In clinical practice, insights into these internal and external factors that influence and play a role in communication and language development are still not brought together too often. Language and communication are seen as the domain of the speech–language pathologist, cognition of the psychologist, motor development of the physiotherapist, and so on. Given the role of all these developmental domains, AAC interventions need an interprofessional perspective. Interventions can only be tailored to the individual needs of the child and their family when all perspectives are integrated in a shared decision-making process [15].

But, how can professionals guide this shared decision-making process for determining goals in AAC intervention for a child with DS? The development of communication and language skills of children with Down syndrome is influenced by a wide array of internal and external factors. For example, ref. [25] found high correlations between language and communication skills and measurements of a wide range of developmental domains in children with Down syndrome: attention, perception, social–emotional development, memory, cognition, orientation and adaptive behavior, and motor development. In a study by Deckers et al. [26], the ICF-CY framework was used to classify contributing factors to communication skills in a multiple case study of six young children with DS; the case used further on in the present paper was part of that study. Within a comprehensive assessment, individual and environmental facilitators and barriers were identified, leading to an individual integrative profile of communication skills. Although the six children with DS shared a developmental and/or expressive vocabulary age and/or level of communicative intent, the children faced similar but unique personal and environmental factors that played an important role in their communication outcomes. Thus, a combination of different factors may lead to the same language outcomes and vice versa, based on a unique pattern of interdependency of ICF-CY domains. Therefore, planning AAC interventions to enhance communication skills in children with DS should be based on a comprehensive view of the competencies and limitations of every individual child andtheir significant communication partners. The following evaluation addresses facilitators and barriers in body functions, structures, activities, participation, and the environment, with a specific focus on individual strengths. But, the question remains: how should we make decisions based on all the available data? This question underlies the development of simulation-based training for professionals and students in health care, paramedic care, and social studies.

### 1.4. Simulation-Based Training

Simulation-based education (SBE) has increasingly been recognized as a useful and safe educational tool in healthcare over recent decades [27], especially to prepare professionals for complex cases. Simulation-based training is defined as ‘an artificial representation of a real-world process to achieve educational goals through experiential learning and is characterized by the use of simulation roles that serve as an alternative for real patients’ [28]. Presently, only a few studies have been published describing simulation-based training programs on interprofessional collaboration [29]; thus, there is a need for continued research in a wide variety of clinical settings to identify which simulation-based interventions are beneficial [27]. We developed a simulation-based training approach for medical and allied health students and their professors (see Section 2.2). In the present study, we describe the experiences with this training. Central to this paper are the questions: What differences can be seen in the process of shared decision-making in an interprofessional team meeting around a child with Down syndrome? and Which factors influence shared decision-making?

## 2. Method

Simulation-based training was provided for medical students and students of allied health professions (e.g., physiotherapists, speech–language therapists, and occupational therapists) from a variety of universities across the globe. The training was provided by the two authors. The professors of the students were present during the training. In an overt ethnographic research design, passive observations were made to ensure careful observations and accurate reporting. After providing the information and the assignment, both authors served as non-participant observers who took field notes. The framework of simulation-based training offered an excellent opportunity to directly experience the behaviors and interactions of a group of people within a specific context. By immersing ourselves in the social setting of interprofessional collaborative teams, we were able to access more authentic information by observing spontaneous interactions. The goal of ethnographic research is not necessarily to test a general theory or hypothesis. Instead, we attempted to gain insight into the group dynamics of an interprofessional team in the context of shared decision-making while we collected rich data.

### 2.1. Participants

In total, over the span of 5 years, 62 groups of 9 to 10 medical and health students from universities in Europe (the Netherlands, Belgium, Germany, Italy, Malta, Sweden, and Finland), the United States (New York and New Jersey), and Asia (the Philippines, China, Hong Kong, and Taiwan) participated in the training. Approximately 85% of the students were female, as often overrepresented in medical and allied health students. They had a variety of backgrounds, experiences working with children with DS, and experiences with interprofessional collaboration. None had previous experience within the context of shared decision-making, as was used in the training. Of the participants, roughly 60% were students of speech–language pathology. Other backgrounds were physiotherapy, podiatry, occupational therapy, psychology, pedagogy, nursing, and medicine. No further personal information about the students was gathered.

### 2.2. Procedure

Participants first received a lecture about interprofessional collaboration, shared decision-making, ICF-CY and the code set of Deckers et al. [26], language development in Down syndrome, and AAC. Although we are well aware of the debate in using the ICF as a framework (i.e., [30]), the fact that ICF-CY codes refer to a well-defined parameter provides a good opportunity to at least “talk about the same” and “use the same language”, which is highly important for interprofessional collaborative work.

Starting the assignment, participants were told to keep the following in mind: In interprofessional collaboration, several involved disciplines formulate shared intervention goals, use the same language, which is accessible and understandable for all involved, and perceive the qualities and perspectives of other disciplines as complementary and valuable. Only in close collaboration can the best health outcomes be accomplished for the individual client [2].

Furthermore, all participants received the following case information with a first reference to ICF-CY codes: Jonas is a 4-year-old boy with Down syndrome who lives at home with his parents and brother for six days a week (e310). One day a week, he stays with a foster family (e340) to relieve his parents from the pressure of taking care of him. His parents and foster parents are involved and motivated to stimulate Jonas in his communicative development (e310). On weekdays, he visits a daycare center that specializes in care for children with multiple disabilities (e360). During daycare, he sees an SLP and a physiotherapist. Sometimes, he sees a doctor and an audiologist (e355). His parents have proposed the following need: “Help us improve the communication of and with Jonas”.

Then, three videos of Jonas, totalling 8 min, were watched together, if possible two times. The videos showed Jonas interacting with his parents at home during a play situation, with the teacher and class assistants during a teaching moment and during snack break, and with peers on the playground. Students were asked to write down detailed observations. After watching the videos, students were divided into groups of 9 or 10. Within each group, every student was assigned a specific role: (1) speech–language therapist, (2) parent, (3) foster parent where Jonas stays for the weekends, (4) audiologist, (5) teacher/day carer, (6) physiotherapist, (7) psychologist, (8) general practitioner, (9) secretary describing decisions and reasoning for these decisions, and (10) student observant describing observations about group dynamics, communication skills, and how and who made decisions. To obtain comparable learning outcomes and to let participants learn about other disciplines, we tried to ensure that every participant was assigned a role outside their professional training. However, it did occasionally occur that a student in speech therapy played the role of the speech–language therapist, given the relatively high amount of participating speech therapy students. When groups contained only 9 members, the roles of secretary and observant were combined. The group was given the task of deciding who would be the chair of the meeting. For a full description of the roles, see Appendix A. Each participant received a description of the case according to his/her role. Some roles shared information, and some roles had unique information. After receiving their information, students had 30–45 min to carefully read and prepare for their role. For example, to look-up what certain codes meant in the ICF-CY when they were not familiar with certain terminology. Students were also allowed to ask questions to the trainers, but only to clarify terminology in their role information. Students were instructed not to share their information with other students before the official start of the interprofessional team meeting since sharing could directly influence the course and the start of the following team meeting. We are aware this does not necessarily reflect a ‘real-life situation’; however, it helped structure the assignments and make it possible to compare groups.

After preparation, students had approximately 60 min for an interprofessional team meeting. The following instruction was given: The goal of the setting is to prepare an interprofessional treatment priority plan to stimulate the communicative development of Jonas. The outcome should include the following: (a) three shared goals for collaborative practice, SMART (specific, measurable, achievable, relevant, and time-bound) formulated, for a period of at least the next 3–6 months, in order of priority; (b) within the interprofessional team, it should be indicated who is responsible for the monitoring and evaluation of goal attainment. How will you monitor and evaluate?; (c) reflection on the group discussion; (d) presentation of your goals and a short rationale (how and why these goals were chosen); (e) presentation of a brief evaluation of the group discussion. Both trainers walked around and observed each group and wrote down their notes. At the end of the sessions, all group secretaries and student observants presented the goals, rationale, and evaluations.

### 2.3. Analysis

All notes of the student groups and trainers were gathered. The first and second authors independently reported and open-coded their observations for meaning. After observing the first twelve groups, we generally refined our codes and constructed categories in themes. All categories and coded observations were compared and discussed until a consensus was reached. After the construction of the categories, we discussed them in relation to the prerequisites for achieving good, efficient, and effective interprofessional collaboration. Saturation for codes and categories was reached during analyses. The Section 3 describes the identified themes and describes several observations per theme. Furthermore, since the goal of the present study was to gain more insight into the dynamics of shared decision-making in an ethnographic way, reflections of the trainers are described based on these observations.

## 3. Results

In this ethnographic research project, we wanted to answer the question of how group dynamics interfere with shared decision-making in interprofessional teams. We identified multiple constructs and themes by passively observing student groups in a simulation-based training exercise. Table 1 describes the categories and themes. For each theme, several observations have been described.

### 3.1. Vision

For most groups, formulated goals were based on strengthening Jonas’ functioning as well as his immediate system. We feel that it is important to take into account the fact that people in Jonas’ environment also have an influence on his overall functioning and that the goals can, therefore, also be focused on the system around Jonas, which indirectly works on Jonas’ functioning. In the group discussions, a treatment or guidance goal was quickly determined, even if the participants did not yet have a holistic picture of the case. We saw that often, after discussing one area of functioning, it was decided to start working on that domain. Interprofessional values and related ethical aspects are an important part of the professional identity of every health or welfare and education professional who wants to work well, safely, effectively, and efficiently with all stakeholders. These values and ethical aspects are personal and stem from the common goal of promoting the health and well-being of a client or a client population. It is also a question of competences to see the clients themselves and their social network as valued team members and experts and to actively involve them in the whole process of treatment or counselling and support. In doing so, the professional realizes that the client population is characterized by diversity.

The fact that certain groups of people have difficulty finding, understanding, and applying information is not solely due to their lack of certain skills. A lot of information, both verbal and written, that professionals and professional organizations share with them is complex and packaged in language that is not easily accessible to them. Think of the jargon of professionals and specialists.

Being able to work together interprofessionally presupposes that you have insight into professionals and other stakeholders from their professional roles and responsibilities and that you can complement each other with the aim of providing person-centered care, support, and guidance. A diversity of roles and responsibilities between team members can be both a resource and a problem for collaboration. Effective teams need this diversity, but everyone in the team must be able to appreciate this diversity. Therefore, this refers to competences with regard to recognizing, acknowledging, and valuing one’s own limitations and strengths, but also those of others, and seeing the need for coordination and cooperation between different professionals, with the common goal of promoting the health and well-being of the client.

### 3.2. Structure

There was often (too) little coordination between those present. Technical jargon, for example, was used relatively often without checking whether it was accessible and understandable to parents or professionals from other disciplines. Paraphrasing or summarizing from time to time can ensure that everyone knows what the discussion is about and can, therefore, have input. Everyone must feel free to ask clarifying questions or ask for more explanation. Interprofessional collaboration means working with and learning from each other.

At the beginning of most roleplays, a chairman was appointed, but our observations indicate that they can and should play a stronger role and ensure that each participant can have their say. In general, the chairman would also be better able to respond to the different personalities present. We did not observe that one discipline was more inclined to take on the role of chairman than another. In some groups, it was decided to give the parent the role of chairman because they know the most about the client and everyone involved. In other groups, the psychologist or general practitioner was chosen as the most ‘natural’ leader of the meeting.

Being able to act interprofessionally also means that professionals have to be good team players. Teamwork competencies refer to any situation in which professionals communicate with each other about and align their actions with the shared goals for the care and support provided to a client. This is about being able to work together in person-centered care, coordinating shared care, solving problems together, and making decisions and choices together. Learning to work in teams means that the professional becomes part of a small, often complex system around a client.

### 3.3. Processes

A professional’s involvement as a team member will often be based on their added value, from their own professional expertise and background, and is complementary to that of other team members. As mentioned earlier, this diversity can also be a source of conflict between team members, for example, regarding who the leader of the team is. It is then important to stick to the client’s goals and how they can be achieved.

The outcome of the interprofessional meeting is strongly influenced by personalities and characters. A person with a strong, present personality may determine the extent to which information is judged and valued as important. For example, if the parent is played by someone with a strong personality, a lot of value is placed on his point of view. However, if the parent remains in the background, his opinions will soon be talked over. This process can occur in any role, but when working interprofessionally, it is important to be extra alert when it comes to the client and their representatives. The chairman of the interprofessional meeting or the care director has a clear task in this.

The order in which symptoms and characteristics are discussed within the interprofessional meeting corresponds strongly to the final prioritization of goals. In various groups, given the size of the group, it sometimes occurred that a certain role could not be taken up, for example, because the audiologist was absent from the conversation. This, of course, had immediate consequences for the way the interprofessional meeting went. Despite the fact that we always provided the same information to all groups, it appears that no group achieved exactly the same three goals or the same prioritization or rationale of goals.

In interprofessional collaboration in multidisciplinary teams, the starting point is the use of a common language and ICF terminology in the role descriptions. However, we did not see this reflected in the target formulation. There was not a single group that linked ICF codes to the formulated goals. What often happened was that technical jargon was used, but clarification questions about essential concepts or ICF codes were not discussed.

Goals were prioritized, but there was not always a clear reasoning as to why the goals were formulated in this order and not in another. It seemed that prioritization was related to the order in which the goals were dealt with in the interprofessional meeting rather than reasoning from the level of functioning of the client. The questions that should be addressed are as follows: Why is this goal so important, and how does it help Jonas to function and participate better? Are there any conditions that we need to take into account in order to achieve this goal?

Problems surrounding Jonas’ hearing were raised by several attendees, but little or no follow-up steps were taken. In a number of these groups, we saw that due to a lack of information, the theme of ‘hearing’ was quickly drowned out by other requests for help, for which the expertise was ‘at the table’. In these cases, it is important that the chairperson, note-taker and/or care director know that there is a need for information and that this must be addressed immediately after the interprofessional meeting. The absence of a role meant that the urgency and importance of the input from this role were insufficiently recognized. This, of course, argues for the presence of all those involved in the interprofessional meeting.

### 3.4. Results

Despite the fact that we always provided the same information to all groups, it appears that no group defined the same three goals or the same prioritization or rationale of goals. Overall, 39 different goals were defined by the groups in different orders of prioritizing and with different time frames or intervention ideas to reach those goals. Although working on interprofessional collaboration and shared decision-making, many goals were connected to one single role, making many goals monodisciplinary instead of interprofessionally. Interestingly, the goal “to improve Jonas’ hearing” was mentioned by 70% of the groups. This was surprising because (a) the parents clearly indicated he experienced no problem with his hearing and did not want Jonas to be tested again, and (b) the role of the audiologist and their total knowledge about Jonas was fairly small when compared to the other role descriptions. Shared decision-making was lacking completely on this point, and all groups chose to convince the parents that Jonas should be tested again. Due to the group dynamics, almost all parents ended up agreeing with the professionals in wording, although their non-verbal communication was not in congruence with that.

Another observation was that in many groups, goals were determined by many different AAC strategies and tools. Some groups advised using manual signs, with or without the combined use of speech (i.e., sign-supported speech), for the communication partners of Jonas. Other groups advised starting to use speech-generating devices or paper-based communication books. AAC advise was strongly steered by the information that was put most strongly to the table by a student playing a specific role. For example, when the psychologist had a more leading role, the cognitive abilities of Jonas were used in decision-making, whereas in other groups, motor development, language development, or vision or hearing status were, to a more or lesser account, used as a rationale for choices of AAC strategies.

In interprofessional collaboration, the starting point should be the use of a common language and ICF-CY terminology in the role descriptions. However, we did not see this reflected in the target formulation. There was not a single group that linked ICF-CY codes to the formulated goals. What often happened was that technical, discipline-specific jargon was used, but clarification questions about essential concepts or ICF codes were not discussed or even raised.

Goals were prioritized, but there was not always a clear reasoning as to why the goals were formulated in this order and not in another. It seemed that prioritization was related to the order in which the goals were dealt with in the interprofessional meeting rather than reasoning from the level of functioning of the client. Questions that should be addressed are as follows: Why is this goal so important, and how does it help Jonas to function and participate better? Are there any conditions that we need to take into account in order to achieve this goal?

In five groups, only eight students participated, and the choice was made to leave the audiologist out of the assigned roles. In these sessions, problems surrounding Jonas’ hearing were raised by several attendees, but little or no follow-up steps were taken. In these groups, we saw that due to a lack of information, the theme of ‘hearing’ was quickly drowned out by other requests for help, for which the expertise was ‘at the table’. In these cases, it is important that the chairperson or note-taker writes down that there is a need for information and that this must be addressed immediately after the interprofessional meeting. The absence of a role meant that the urgency and importance of the input from this role were insufficiently recognized. This, of course, argues for the presence of all those involved in the interprofessional meeting.

Of course, we also observed many positive behaviors in all constructed categories and themes. Positive behaviors reflect the opposite of the examples described in Table 1. For example, we observed some groups where, from the start, the perspective of the parents was set as leading the discussion. Parents in these groups were often asked if they still understood and if what was discussed was in the best interest of Jonas according to their perspective. Additionally, there were groups with a strong or stronger chairman, making sure that all roles could share their information and, most importantly, that conflicting information was discussed. Some groups agreed to try not to use any jargon.

## 4. Discussion

The motto of shared decision-making is ‘Without me, there is no decision about me’. However, communication with a client, especially in the healthcare sector, is still often approached from the perspective of the professional and is sometimes characterized by a paternalistic communication style, in which decisions are mostly based on the information and opinions of the professional [31]. As a result, clients usually feel unheard and excluded from the decision-making process, especially when decisions are made in a team. An important condition for shared decision-making is the willingness of the professionals involved to take the client’s vision and choices into account. In many groups, we saw this paternalistic communication style of students playing a professional role in that they mostly tried to argue with and convince parents that a goal should be set based on their role-specific information. Explaining to the parents what professionals have determined is very exclusionary and condescending. In many groups, students playing the role of parents did not feel included in the decision-making process.

An important aspect of shared decision-making is that the client really understands what it is about and what a decision will mean for him and his personal situation. We did not observe this in many groups, leaving parents and many of the professionals at the table as well, confused about what decisions would mean for their daily life. Professionals often use technical terms and complex concepts and do not actively check whether their client has actually understood the information [32]. This may result in parents or other professionals who are not familiar with discipline-specific jargon taking a passive role in the process of shared decision-making, which might be misinterpreted as disinterest: the client apparently does not want to ‘decide together’ or leaves it up to the professional [33]. The passive role of parents may be well understood by their experience, which is repeated time and again only for them to be outnumbered or overruled in these discussions. With the reclamation method, sometimes also called the retelling method, the professional can check whether the information has come across correctly [2]. Does the client know all the pros and cons of the different options? The professional can do this by asking the client to describe in their own words what has just been discussed. The professional can then make adjustments or supplement if necessary. It is important that the client does not get the feeling that they are being monitored, so the professional should relate the question to them as much as possible: ‘I would like to know if I have explained it correctly. What are you going to tell (or do) at home?’.

Developing basic communication skills is a competency that is covered in all health, welfare, and teacher education and training programs, but most students and professionals often have less knowledge and experience with interprofessional communication [2]. Interprofessional communicative competencies are important for collaboration with professionals from other disciplines and with clients and their social networks. This involves, for example, learning to speak each other’s discipline-specific language and tuning into your conversation partners, diminishing the amount of technical jargon used. Diversity, in the broadest sense of the word, also plays a role in this coordination. The use of jargon is often seen as a barrier to effective collaboration. Providing information that is accessible and understandable to everyone, including the client and the social network, contributes to safe and effective interprofessional care.

As we can clearly see from our experiences with simulation-based training, shared decision-making is thus an ongoing challenge, especially in an interprofessional context. Sharing knowledge and experiences during a decision-making meeting does not guarantee that team members will continue to work together afterwards [10]. Each team member will make their own personal interpretation of the situation based on the information provided by the others and then make their own prioritization regarding the steps in the care and support process [2]. This means that professionals must be able to deal with the perspectives of the client himself, his social network, and other professionals and that they have to adjust their own perspective accordingly. Only when a common perspective has been reached through communication with agreement on the prioritization of advice can we really speak of shared decision-making in the interprofessional context. As can be expected, most of the groups in our training did not reach this stage after following an extensive lecture and the current assignment, which strongly suggests the need for continued education in health and social care interprofessional collaboration skills. However, using the current assignment and reflecting with the students afterwards can be seen as a valuable starting point in raising awareness and gaining more insight into the required competencies for interprofessional collaboration. Therefore, we advise integrating the developed assignment into a broader interprofessional education program, which should focus on the core competencies of interprofessional collaboration as proposed by the Interprofessional Education Collaborative [34].

Each team member, including the client and the people from his social network who are important to him, has his or her own specialist knowledge and experiences that are relevant to the specific situation and that can contribute to making informed decisions [10]. Motivating and challenging a team to discuss conflicting ideas and perspectives (including those of the client and their social network) and to transform them into a single perspective will benefit the client’s care, education, and support. But, how do you arrive at a well-founded decision in which all this knowledge and perspectives have been weighed and in which everything is integrated? Makoul and Clayman [35] formulated nine essential steps for shared decision-making. Some of these elements of Makoul and Clayman still veer too much towards a paternalistic style, such as making a recommendation, because this should really only be conducted at the explicit request of the client. Therefore, we prefer the four steps for shared decision-making of Stiggelbout et al. [36]. We supplemented the steps proposed by [2] from an interprofessional perspective:

1. The professional informs the client and/or his colleagues that a decision has to be made and that everyone’s opinion is important and will be discussed. The professional emphasizes that the goal of the conversation is to make a choice (creating choice awareness).
2. The professional explains the different options and possibilities and discusses the advantages and disadvantages of each option. In doing so, he leaves room for any additions from others present. The pros and cons are substantiated with possible scientific evidence.
3. The professional and the client discuss the client’s preferences, which are linked to norms and values, and the professional supports the client in weighing up options. In this way, everyone can indicate what is important to them. It is also important to relate the pros and cons to the client’s individual situation, e.g., social, home, and work situation. Keep in mind that different norms and values may play a role for clients or professionals with different cultural backgrounds. The professional must provide room to discuss this.
4. The professional and the client discuss the client’s desire and possibilities to make an informed decision. They then make the decision together or postpone it so that, for example, the client can think about it or discuss it with family and friends. There is also talk of a follow-up to the appointment and the decision. For some clients, such as the elderly or people with a different cultural background, they may feel uneasy participating in decision-making. They may assume that the professional is the one who knows what they are doing and that the choice will come from them. Therefore, it is important to explain that the professional does indeed know what he or she is doing but that it is also necessary to know what is important to the client. It may be that after discussing preferences, the client chooses to leave the decision to the professional. This is also a shared decision if the client actively indicates this.

The chair of the interprofessional meeting is responsible for leading the meeting well, regulating the conversation, introducing new topics, and, above all, completing the assignment items of the meeting in a good way. The chair makes sure that the meeting starts on time, maintains order, and makes sure that everyone who wants to say something has their say and that there is not too much talking. Additionally, of course, they pay close attention to the time, so that the goal of the discussion is reached within the given timeframe. The discussion has a specific process-based objective. The most important task of the chairman is to achieve this objective. Particular attention should be paid to the proper completion of the discussion. In order to show this necessary leadership in interprofessional communication Nieuwboer et al. [37] suggested to (1) ensure that all team members are connected and motivated; (2) help develop a shared vision; (3) foster a culture in which the team members empower each other as much as possible; (4) work from connection and not from hierarchy, but with a certain degree of authority and expertise; (5) train team members to deal well with conflicts; (6) ensure team members to articulate their expectations and needs. Based on the results of this study, more interprofessional education is necessary for students to play this role in their professional lives.

### Limitations

A limitation of simulation-based training and the assumption of roles we do not typically perform is that there is no chance for social relationships to develop over time, which can enhance or interfere with interprofessional collaborative practice. Where clients tend to have a taken-for-granted trust relationship with healthcare professionals to provide a competent service that meets their needs, trust is challenged after the introduction of changes in the organization and performance assessment of health professionals and in public attitudes to health care science [38]. In this simulation-based role play, building trusting relationships with the other professionals or the clients was extremely limited by the lack of time for the simulation. Students were put together in groups, who often did not know each other. This may have interfered with how students played their roles and might have affected the group dynamics and outcomes of the assignment.

Simulation-based training has been conducted throughout the world. In the present study, we combined all our observations. It might be that cultural background played a role in group dynamics. We did not check for the cultural sensitivity of the training per se. We discussed the assignment beforehand with university staff and did not encounter any comments about culturally insensitive information. Many groups of participants were indeed composed of different cultural backgrounds. Cultural background might influence the role somebody takes in a group discussion, so the cultural background of participants might have influenced the way how they played their roles. We did not study this in further detail. Thus, our results might be influenced by cultural backgrounds; however, we did not observe substantial differences between group discussions and outcomes for different participants across the world. In a follow-up study, we advise taking the cultural background and communication styles of students into account.

Any simulation-based training is a simplification of a real-life situation. Information in the current assignment was scripted, and conflicting information was added to the roles to elicit the required discussion. This may not directly mirror a real-life case. Collaborating with different professionals and with parents is complex and is something that students need to learn by experience. Of course, they have to gain these experiences in real-life as well, for example, during internships. However, this kind of simulation-based training does give universities the opportunity to let students experience interprofessional collaboration in a relatively controlled and ‘safe’ environment. They have to learn how to form group meetings, how to bring information to the table, and how to take on their roles and responsibilities, all for the greater purpose of good care. Given the experiences with the current assignment, simulation-based training seems like a wonderful teaching moment. The themes and examples, as described in Table 1, were also discussed afterwards with students to help their learning experiences further.

## 5. Conclusions

The outcome and goalsetting of an interprofessional meeting with a client or their parent(s) is highly influenced by group dynamics, as was experienced using simulation-based training with participating students from a range of backgrounds in multiple countries around the globe. The vision, structure, process, and results of the meeting are affected by multiple inter- or intrapersonal factors, like shared decision-making or communication skills.

The materials and case used in the present study can be used by other universities to make their students aware of what interprofessional collaboration and shared decision-making entail. We advise that the current training is embedded in a larger educational module about interprofessional collaboration, given the many competencies in interprofessional collaboration that students should work on [34], like role clarification, interprofessional communication, dealing with interprofessional conflicts, how to make shared decisions, functioning in a team, and collaborative leadership.

## Figures and Tables

**Table 1:** Categories, themes, and observations.

Constructed Categories (In a Random Order) and Themes	Observations
**Vision**	
Collaborative leadership	Above all, there was a lot of monodisciplinary thinking, e.g., I want to have my say and this is MY expertise. This led to students wanting to share all information about their role first without asking for and integrating the information of others. In multiple groups, parents had to say: “please listen to my ideas, realize that as a parent I am also an expert when it comes to Jonas”. When other roles shared their information, especially in a lengthy manner, other students seemed distracted from the discussion; they did not feel responsible for what the other person said and failed to integrate this into their own thinking, looking away. As a result, shared responsibility was hardly established. Many formulated goals were not directly related to Jonas’ functioning but rather to how the people around him could learn or do things differently, showing the idea that students were aware of their own roles in establishing better communication outcomes for Jonas.
**Structure**	
Team functioning	In most groups, a chairman was only appointed after the researchers intervened. This means that in most groups, there was no structured (start of the) discussion and nobody made sure that all roles had their say, or that at least the parents did. The structure of the discussion was lost and the timekeeping observed was inappropriate. At the end of the 60 min, in at least 10 groups, not all students had shared all relevant information before decisions on goals were made. Responsibility for the evaluation and monitoring of goals was missing. In the discussion, goals were formulated which are not appropriate to the communication level of the child: “Jonas is able to communicate with his friends within three months” (at the moment, he had almost no contact with children of his own age). No one in the discussion intervened. Students failed to take cultural differences into account. As supervisors, we were very direct because we intervened and gave direct feedback. Many students were not used to this. In the team, they themselves did not often directly addressed each other’s feedback points. Being kind to each other was mainly due to monodisciplinary thinking and pigeonholing: ‘This is my piece and that’s your piece’.
**Process**	
Role clarification	Questions like: What does an audiologist do? or What could or should be your role in the intervention plan? were often not asked. During evaluations, it was often not clear to students ‘why’ a certain role was or was not involved. Only 30% of the groups started with a round of personal introductions and role-clarification. Thus, 70% of the groups started directly with discussing content and information about Jonas without mentioning their specific role. The chair of the meeting was not always appointed, or the role was not performed well. Questions like “Who is responsible for evaluation and monitoring?” were not asked or discussed. Professionals assumed that parents were aware of the role and responsibilities of the professionals present. At the same time, most parents indicated that they did not know what the involved professional roles represented. Professionals did not re-introduce each other during the roleplay (assuming that the parent was able to remember), which led to parents not knowing what information came from which role at the end of the meeting.
Dealing with interprofessional conflicts	Students adhered to their own information and views in discussions. Some examples are as follows: “I was the audiologist and I had important information, so one of the three goals had to be set about hearing”. “I see that he has difficulty eating, so he needs to be trained because he has an eating problem” (despite the fact that the case description indicated that there is no problem). No discussion of nutrients and calorie intake; mum was proud of Jonas being able to eat banana for the last three weeks. “He needs PECS (i.e., Picture Exchange Communication System), because he can do something with pictograms according to my background information” (but there was also a vision problem; information was not integrated into one shared perspective of the functioning of Jonas). In the event of conflict, raising one’s voice and seeking confrontation was observed in order to gain one’s own point of view. If parents want something that does not seem sensible to professionals or that is not feasible, then the professionals need to provide arguments as to why. This was hardly ever done. Every group of professionals tried to convince the parents of their beliefs (without accurate argumentation or exchange of perspectives). Participants immediately defended themself in the event of feedback or questions about purpose and rationale. Students kept talking about conflict; it could not be set aside, but it was always referred back. There was a lack of group discussion techniques, relating to the lack of a clear chair for the meeting.
**Results**	
(Shared) decision-making and goal-setting	Parents got their turn somewhere in the conversation, but only a small number of groups started with the parent. Parents were not asked for their wishes; there were little to no patient-centered discussions. Experiences of other professionals were not questioned to parents: ‘do you see this at home the same way?’ Test results were mainly the starting point to base decisions on. ICF-CY codes were not mentioned. Often, only the written information was used in discussions, not the table with ICF-CY codes in role descriptions. Many goals were determined by one or multiple professionals. The needs of (foster) parents were often not met. Parents felt left out of the discussion or announced afterwards that they did not understand all information given by the other roles. Therefore, they did not feel part of the shared decision-making. However, in several groups, parents did feel part of the shared decision-making. This was often the case when the student playing the parent had a strong personality or when there was a chair who often turned to the parent to ask if they understood and agreed.
Interprofessional communication	Speaking in technical jargon, not adapting language to, e.g., parents: “Jonas has a score of three standard deviations below the norm”. Talking about “him” instead of naming him: “Jonas”. In some groups, “the child” was also heard. Although parents were part of the discussion, in some groups, professionals were talking about “they” when expressing issues about (foster) parents. Halfway through, parents indicated that they had not understood what the discussion was about so far. Parents reported that it took courage and time to speak up so they did not understand all the information. At the end of the discussion, professionals said: “And now we’re going to explain to parents what we’ve determined”. In some groups, no clarification questions were asked to each other. In other groups, a lot of clarification questions were asked, which also took a lot of time out of the discussion to reach shared goals.

## Data Availability

Data are available on students’ evaluation of their reflections on interprofessional competencies. No publicly archived datasets were analyzed or generated during the study.

## Appendix A. Role Descriptions

### Appendix A.1. Speech–Language Therapist

- Due to a medical condition, he suffered from intestinal obstruction and subsequent problems in drinking and eating. He underwent surgery, from which he recovered well. Given his sustaining problems in drinking and eating, he started SLP therapy with you when he was 6 months old. Drinking and eating are no longer an issue (d550, d560).
- He failed at an auditory discrimination task in which you asked him to point to the correct picture. This task is to gain insight in whether a child can hear the difference between words that sound almost the same (minimal pairs). The task was ‘car… bar, where is car?’ A picture of a car and of a bar were presented to him.
- You administered the Communicative Development Inventories, a questionnaire to determine the number of words a child understands and uses. These are the results:
  ∘ Parents: receptive vocabulary age 1;10 years (i.e., one year and ten months), expressive vocabulary age 1;5 years;
  ∘ Teacher: receptive vocabulary age 1;4 years, expressive vocabulary age 1;2 years;
  ∘ SLP: receptive vocabulary age 1;8 years, expressive vocabulary age 1;3 years.
- You are training Jonas in using graphic symbols (picto’s). Although this is often not an effective way for him to communicate, you really see the advantages of working with these symbols. He understands what some of them mean, and you use them to give him insight into the schedule of activities during your therapy session. You might have to convince the others to work with graphic symbols.
- Severe impairment in the reception of spoken language communication partners. Do not only talk to him!

**Table A1:** Overview of ICF-CY codes for the role of speech-language therapist.

d3 Communication	
d310–d329 COMMUNICATING—RECEIVING	
d3100 Responding to the human voice	-
d3101 Comprehending simple spoken messages	-
d3102 Comprehending complex spoken messages	--
d3150 Communicating with—receiving—body gestures	0
d3151 Communicating with—receiving—general signs and symbols	-
d3152 Communicating with—receiving—drawings and photographs	-
d320 Communicating with—receiving—formal sign language messages	-
d325 Communication with—receiving—written messages	--
d330–d349 COMMUNICATING—PRODUCING	
d330 Speaking	--
d331 Pre-talking	--
d332 Singing	?
d3350 Producing body language	+
d3351 Producing signs and symbols	--
d3352 Producing drawings and photographs	--
d340 Producing messages in formal sign language	-
d345 Writing messages	--
d350–d369 CONVERSATION AND USE OF COMMUNICATION DEVICES AND TECHNIQUES	
d3500 Starting a conversation	--
d3501 Sustaining a conversation	--
d3502 Ending a conversation	--
d3503 Conversing with one person	--
d3504 Conversing with many people	--
d355 Discussion	--
d360 Using communication devices and techniques	NA

Note. Activities and Participation: 0 = no problem; - = mild (1) to moderate (2) problem; -- = severe (3) to complete (4) problem, + = relative strength, ? = inconclusive.

### Appendix A.2. Physiotherapist

- I just do the exercises with him, but I do not communicate with Jonas much. He uses some signs, but I do not know what he means by them. They are not the regular signs I learned during a course from our SLP 2 years ago.
- His mobility skills are relatively well-developed. He can walk by himself and is able to step over small objects. Climbing the stairs is harder for him.
- At the moment, I am working on his fine motor skills, which are also relatively well-developed. The most important goals are his self-help skills, so that he, for example, can go to the toilet by himself. His fine motor skills are not fully developed, so he can (un)button and (un)zip his pants by himself.
- I think he is motorically capable of using manual signs, but he may be too lazy and sloppy to always execute them precisely.
- I notice that his attention is better when I hold him or do the exercises with him. When he is jumping on the trampoline, his attention is better, and he responds more quickly.

**Table A2:** Overview of ICF-CY codes for the role of physiotherapist.

d4 Mobility	
d4153 Maintaining a sitting position	+
d450 Walking	0
d4550 Crawling	NA
d4600 Moving around within the home	+
d4601 Moving around within buildings other than home	+
d4602 Moving around outside the home and other buildings	+

Note. Activities and Participation: 0 = no problem (0); - = mild (1) to moderate (2) problem; -- = severe (3) to complete (4) problem, + = relative strength.

### Appendix A.3. Teacher

- I have concerns about his hearing functions. Especially when addressed in the classroom, he often does not respond. Additionally, when I ask him again and again, he will not respond.
- Jonas uses some signs; he does not speak, but they are often not clear to me. When I ask him to repeat, he does not respond. I do not think he has the motor skills to learn clear signs. What does the physiotherapist think?
- Jonas has no contact with peers, he often walks by himself on the playground. Should we do something about this? I do not think I will have the time during the break to help him play with other children.
- On Monday morning, he is often very tired. When asked what he did during the weekend, he is not able to answer. When I ask him, did you go to…? (parents write notes to the teacher with stories from the weekend), he often nods yes. Other questions he cannot answer. I really want him to tell the class about his weekend.
- I followed a course in sign language from our SLP 2 years ago.
- He is often distracted. I want to work on his ability to focus longer. When doing a group assignment, he always wanders off. How should I work on that?
- He really gets upset when the SLP or the physiotherapist walk in to get him for a therapy session. Although I just told him 10 min ago they would come.

**Table A3:** Overview of ICF-CY codes for the role of teacher.

d130–d159 Basic Learning and Acquiring Knowledge	
d130 Copying	0
d1310 Learning through simple actions with a single object	-
d1311 Learning through actions by relating two or more objects	--
d1313 Learning through symbolic play	--
d1314 Learning through pretend play	--
d132 Acquiring information	--
d1330 Acquiring single words or meaningful symbols	--
d1331 Combining words into phrases	--
d1332 Acquiring syntax	--
d135 Rehearsing	--
d1370 Acquiring basic concepts	--
d1371 Acquiring complex concepts	--
d1400 Acquiring skills to recognize symbols	--
d1401 Acquiring skills to sound out written words	--
d1402 Acquiring skills to understand written words and phrases	--
d1550 Acquiring basis skills	+
d1551 Acquiring complex skills	--
d160-d179 APPLYING KNOWLEDGE	
d160 Focusing attention	--
d1600 Focusing attention on human touch, face and voice	-
d1601 Focusing attention to changes in the environment	--
d1608 Focusing attention, otherwise specified: to interesting objects	+
d163 Thinking	--
d1630 Pretending	--
d166 Reading	--
d170 Writing	--
d175 Solving problems	--
d177 Making decisions	--

Note. Activities and Participation: 0 = no problem (0); - = mild (1) to moderate (2) problem; -- = severe (3) to complete (4) problem, + = relative strength.

### Appendix A.4. Doctor/General Practitioner

- At birth, duodenal atresia was identified, which is the congenital absence or complete closure of a portion of the lumen of the duodenum (s5408). Due to this medical condition, he suffered from intestinal obstruction and subsequent problems in drinking and eating. He underwent surgery, from which he recovered well. Given his sustaining problems in drinking and eating, you referred him to a speech–language pathologist. He started SLP therapy when he was 6 months old.
- Foster parents called you because of frequent mid-ear infections and colds. This seems to impair his hearing at times. You have called in the audiologist who screened the hearing of Jonas twice before.

Only after the audiologist has explained the impact of frequent mid-ear infections: how would you cure this? No medication should be used. If a hearing screening determines a loss of at least 25 dB in the best ear, and the infections are indeed often recurrent, grommets (tubes in the ear) may be a solution. This would mean a new hospitalization. Is that a problem?

- I got a report from the ophthalmologist. Jonas has severe problems in his visual acuity functions (b2100) (sensing form and contour, both binocular and monocular, for both distant and near vision) and mild problems with his quality of vision (b2101) (Seeing functions related to the entire area that can be seen with fixation of gaze), however, he is not wearing glasses (e1251). You want to have an answer to the following questions: What does everybody experience regarding Jonas’s vision? Should we consider glasses?

### Appendix A.5. Parent

- He can understand spoken language, but lots of repetition of the message is needed.
- I most often use spoken language, but for the most important words, I will also provide him with a manual sign (sign-supported speech).
- I really think the classroom is a wonderful place for him to develop. All the other children are of the same developmental age, and given the social skills of Jonas, I think he will do well in contact with other peers. Making friends will benefit his skills.
- No concern about his hearing functions. When I tell him something, he always responds. He sometimes has a cold, but nothing to worry about. When the doctor mentions hearing screening: I overheard the doctor talking about a new hearing screening. I do not want that; it is too much for Jonas.
- I really want him to be able to tell about what he did at school, during SLP, or at his foster parents. Is there anything, an app, for example, that we can use to do this?
- He can speak some words which I can understand very well, but I can imagine that not everybody would understand. What strikes me is that sometimes he pronounces a word correctly, for example, ‘dog’, and suddenly it looks like he forgot how to say it, and he says ‘dod’ or ‘do’. Should we work on his speech? Should I correct his unclear speech and provide the correct word?
- He can sit and watch the television for an hour. Well, actually, he does not sit. He stands close to the screen. Why does he do this? If I tell him to sit down, he always returns to the TV in a few seconds.

### Appendix A.6. Foster Parent/Weekend Care Person

- You have to give Jonas time to respond. If you ask him too many questions, he will get confused and irritated. When we ask him something, we wait for a while. Sometimes, we repeat the question, but not too often.
- Concern about his hearing functions. When I call him, he often does not respond. His ears look red at times. He often has a cold.
- He is with us on Sundays. We always go out on trips. He really likes the zoo. We have been there about 20 times now. When we walk by the elephants, I always make the manual sign and the sound of an elephant. After the first time, he started imitating me, and now he uses the manual sign and sound spontaneously when we walk by the elephants. Should we do this with more animals? One at a time, or just every animal? Additionally, what about other topics, should we practice this with him in the supermarket as well? I think he is able to learn this, so I will work on this with him.
- We really have to keep him busy. We cannot just let him play by himself because he goes wandering off. While playing, I have to point to objects or talk about them. Jonas does not take initiative in play. He is only interested in some toys!

### Appendix A.7. Audiologist

- At neonatal hearing screening, no problems with his hearing (b1560) or with his ear structure (s240, s250, s260) were indicated. Often, people say that most children with Down syndrome suffer from hearing problems, but recent studies show that, at birth, about 80% of children have normal hearing levels.
- At age 2.5, no problems with his hearing were indicated
- His next appointment is in a year. Why did you ask me in?
- Only after you have been explained why you were called in: explain the effect of frequent mid-ear infections on hearing functions to the parents and foster parents (adapt your language to them!) and why they should be aware of this. Frequent mid-ear infections cause Jonas’ hearing levels to fluctuate from time to time. His hearing is sometimes better than at other times. This means that when he does not have an infection, he clearly hears better than when he does. This has an effect on his ability to learn and speak words correctly since his phonological awareness is affected by this.

How can they detect an infection (red, fluids, fluctuating hearing levels, different behaviors in responding to verbal questions)? Would you now consider a new appointment?

**Table A4:** Overview of ICF-CY codes for the role of audiologist.

b230–b249 Hearing and Vestibular Functions	
b230 Hearing functions	-
b2300 Sound detection	-
b2301 Sound discrimination	--
b2302 Localization of sound source	0
b2304 Speech discrimination	0
b235 Vestibular functions	0

Note. Activities and Participation: 0 = no problem (0); - = mild (1) to moderate (2) problem; -- = severe (3) to complete (4) problem, + = relative strength.

### Appendix A.8. Psychologist

- Just administered the Baileys Scale of Infant Development (test for developmental age). At age 4;9 years, his developmental age is around 1;9 years (mental scale) and around 2;3 years on the non-verbal mental scale [years;months]. Short observations:
  ∘ He hesitates before entering the room, but after taking my hand, it is OK. During the second time of testing, he walked to the chair by himself without having to ask him.
  ∘ In the beginning, he is cooperative and responds well to my questions, but after a few minutes it looks like he is somewhere else. When the assignments are getting harder, he loses focus on the task and is distracted. His body language gives me particular insight. He does not respond to verbal instructions anymore at that time. How do the teacher and SLP cope with this? What do the parents and foster parents think about this? Additionally, what about the physiotherapist?
  ∘ Attention is a lot better when he is playing with interesting toys. Do you also see that in the classroom or during therapy?
  ∘ He is able to match objects on color, up to a maximum of three colors.
  ∘ He needs the time to understand what you are asking of him. Needs a lot of repetition.

**Table A5:** Overview of ICF-CY codes for the role of psychologist.

b140–b189 Specific Mental Functions	
b1400 Sustaining attention	--
b1402 Dividing attention	-
b1440 Short-term memory	--
b1442 Retrieval and processing of memory	-
b1560 Auditory perception	?
b1561 Visual perception	-
b1564 Tactile perception	0
b1568 Perceptual functions, otherwise specified: sensory integration	--
b1600 Pace of thought	--
b163 Basic cognitive functions	--

Note. Activities and Participation: 0 = no problem (0); - = mild (1) to moderate (2) problem; -- = severe (3) to complete (4) problem, + = relative strength, ? = inconclusive.

### Appendix A.9. Secretary

- Make notes of the discussion.
- Write down shared goals and rationale.
- Evaluate the process of communication between professionals and parents.
